# Longitudinal biomarker studies in human neuroimaging: capturing biological change of Alzheimer’s pathology

**DOI:** 10.1186/s13195-025-01920-6

**Published:** 2025-12-04

**Authors:** Larissa Fischer, Dana Parker, Samira Maboudian, Corrina Fonseca, Claudia Tato-Fernández, Lucie Annen, Prithvi Arunachalam, Julia R. Bacci, Michelle Barboure, Serena Capelli, Stamatia Karagianni, Lyduine E. Collij, Paul Edison, Nick C. Fox, Nicolai Franzmeier, Michel J. Grothe, William J. Jagust, Anne Maass, Maura Malpetti, Ross W. Paterson, Aitana Sogorb-Esteve, Michael Schöll

**Affiliations:** 1 German Center for Neurodegenerative Diseases (DZNE), Magdeburg, Germany; 2 Department of Neurobiology and Behavior, University of California, Irvine, USA; 3 Department of Neuroscience, University of California, Berkeley, USA; 4 Turku PET Centre, University of Turku, Turku University Hospital, Turku, Finland; 5 Division of Geriatric Psychiatry, University Hospitals of Geneva, Thônex, Switzerland; 6 Department of Psychiatry, University of Geneva, Geneva, Switzerland; 7 Department of Radiology and Nuclear Medicine, UMC Vrije Universiteit Amsterdam, Amsterdam, The Netherlands; 8 Amsterdam Neuroscience, Brain Imaging, Amsterdam, The Netherlands; 9 Department of Epidemiology and Prevention, Wake Forest University School of Medicine, Winston-Salem, USA; 10 Alzheimer Center, Department of Neurology, UMC Vrije Universiteit Amsterdam, Amsterdam, the Netherlands; 11 Bioengineering Department, Istituto di Ricerche Farmacologiche Mario Negri IRCCS, Ranica, Italy; 12 Wallenberg Centre for Molecular and Translational Medicine, University of Gothenburg, Sahlgrenska University Hospital, Gothenburg, Sweden; 13 Department of Psychiatry and Neurochemistry, Institute of Neuroscience and Physiology, The Sahlgrenska Academy, University of Gothenburg, Gothenburg, Sweden; 14 Clinical Memory Research Unit, Department of Clinical Sciences, Faculty of Medicine, Lund University, Malmö, Sweden; 15 Division of Neurology, Department of Brain Sciences, Faculty of Medicine, Imperial College London, London, UK; 16 School of Medicine, Cardiff University, Cardiff, UK; 17 Dementia Research Centre, UCL Queen Square Institute of Neurology, University College London, London, UK; 18 UK Dementia Research Institute at UCL, University College London, London, UK; 19 Institute for Stroke and Dementia Research (ISD), University Hospital, LMU Munich, Munich, Germany; 20 Munich Cluster for Systems Neurology (SyNergy), Munich, Germany; 21 Reina Sofia Alzheimer Centre, CIEN Foundation, ISCIII, Madrid, Spain; 22 Faculty of Natural Sciences, Otto Von Guericke University Magdeburg, Magdeburg, Germany; 23 Department of Clinical Neurosciences and Cambridge University Hospitals NHS Trust, University of Cambridge, Cambridge, UK; 24 UK Dementia Research Institute at University of Cambridge, Cambridge, UK

**Keywords:** Alzheimer’s disease, Biomarker, Longitudinal, Neuroimaging, PET, MRI

## Abstract

Despite extensive research, open questions about the biological underpinnings of Alzheimer’s disease (AD) remain. Neuroimaging biomarkers based on positron emission tomography (PET) and magnetic resonance imaging (MRI) offer in vivo insights into these complex biological changes and interactions. However, most evidence to date comes from cross-sectional studies, limiting our understanding of disease progression. Longitudinal studies enable the investigation of biological changes within individuals, revealing how pathology evolves over time. With this review, we provide an overview of how longitudinal imaging biomarker studies have advanced the field and how they can contribute to future research. We highlight longitudinal biomarker studies that have provided critical insights into disease trajectories, staging, and individual variability. We further assess longitudinal multimodal studies which have elucidated interactions between AD-specific pathology, amyloid-β and tau, and broader biological changes like neurodegeneration, neuronal dysfunction, vascular disease, and inflammation. Further, we discuss associations of brain changes with symptomatology and clinical outcomes and conclude with challenges and future directions.

## Introduction

Alzheimer's disease (AD) is characterized by a cascade of biological changes, particularly the accumulation of amyloid-β (Aβ) and tau pathology, which progressively affect neuronal functioning and integrity. Pathology impairs cognitive abilities and eventually leads to AD dementia. Insights into the pathophysiology of AD have been gained from a variety of biomarkers, with human postmortem histopathological studies as the gold standard. A biomarker is “a characteristic that is objectively measured and evaluated as an indicator of normal biological processes, pathogenic processes, or pharmacologic responses to a therapeutic intervention” [1]. In recent decades, neuroimaging biomarkers have been developed using positron emission tomography (PET) to assess Aβ and tau burden, as well as PET and magnetic resonance imaging (MRI) to evaluate nonspecific pathophysiological changes such as neurodegeneration, network dysfunction, vascular disease, and inflammation [2, 3].

Human neuroimaging has the important benefit of providing region-specific insights into changes of the underlying biology in an in vivo setting, but the field has been largely dominated by cross-sectional studies. Consequently, most of the evidence for the proposed model of AD pathophysiological sequences has relied on cross-sectional imaging or histology studies. However, as the field has grown, information from longitudinal and multimodal imaging studies is becoming increasingly available. With repeated imaging over time, those studies can provide a more detailed understanding of the temporospatial development of AD, uncover mechanisms contributing to individual variability in disease trajectories, and can further help to narrow down clinically relevant biomarkers. These studies not only allow inferences about the dynamics of disease progression, but also help to identify potential underlying mechanisms affecting the disease process and outcomes. When incorporating interventions, these studies are further uniquely poised to identify causal relationships and can provide insights into how the brain reacts to novel therapies.

The purpose of this review is to assess the utility of longitudinal neuroimaging studies in capturing the biological changes along the AD continuum and their interactions with each other. First, we will assess what aspects of the underlying pathology are captured by human neuroimaging. We will then examine what longitudinal imaging biomarkers can tell us about (1) the disease trajectory and pathological staging in AD, (2) relationships between AD-specific pathology and other nonspecific biological changes observed in AD (neurodegeneration, neuronal dysfunction, vascular disease, and inflammation), and (3) how these interrelated biological changes are linked to symptomatology and clinical outcomes. Finally, we conclude by discussing potential imaging biomarkers that currently lack longitudinal support, highlighting challenges and possible insights from future studies. By focusing on the advances and challenges in longitudinal imaging biomarkers of AD, this review ultimately aims to provide insights into the biological underpinnings of AD that could contribute to improved tools for diagnosis and disease monitoring, as well as determining suitable treatment targets to attenuate AD progression.

## Disease trajectory and pathological staging

First, we provide a brief overview of AD pathology and imaging biomarkers before discussing longitudinal trajectories and staging. AD is associated with diverse pathological changes that can be captured with neuroimaging methods in humans, each reflecting distinct yet interacting biological processes. The core AD features, discovered in postmortem histological research, are extracellular Aβ plaques and intraneuronal tau neurofibrillary tangles [4–7]. Both can be measured in vivo using PET imaging, which captures the buildup of these protein aggregates in the brain [8, 9]. Further, nonspecific pathological changes related to AD encompass neurodegeneration and neuronal dysfunction, including changes in brain metabolism and networks, and changes related to vascular disease and inflammation. An overview is provided in Table 1.

**Table 1:** Imaging of biological changes related to Alzheimer’s disease

Pathological change	How can we measure this change with imaging?	What underlying biological feature or process is targeted?
Amyloid-β (Aβ) accumulation	Positron emission tomography (PET) imaging using, for example, the tracers [11C]Pittsburgh compound B (PiB), [18F]florbetaben (FBB), [18F]florbetapir (FBP), and [18F]flutemetamol [8–10]	Insoluble Aβ plaque accumulation surrounding neurons is a specific feature of Alzheimer’s disease (AD) and can be assessed by specific tracers binding to these proteins [6, 7]
Tau accumulation	PET imaging using, for example, the tracers [18F]flortaucipir (FTP), [18F]PM-PBB3/florzolotau, [18F]MK-6240, and [18F]PI-2620 [11, 12]	Tau neurofibrillary tangle accumulation within neurons is a specific feature of AD and can be assessed by specific tracers binding to tau aggregates [4, 5]
Neurodegeneration	T1- and T2-weighted structural MRI (sMRI) focusing on the gray matter volume of brain structures, FLAIR sequences, and diffusion-weighted imaging (DWI) focusing on white matter microstructural connectivity	Brain atrophy, white matter hyperintensities (WMH), and white matter impairment are disruptions commonly found over the course of AD. Hippocampal atrophy assessed with sMRI is a key prognostic feature of AD [13]. Widespread abnormalities in white matter microstructure have been consistently reported in DWI studies of patients with AD [14]
Neuronal dysfunction	Fluorodeoxyglucose (FDG) and synaptic vesicle protein 2 A (SV2A) PET using the [11C]UCB-J or [18F]SynVesT-1 tracer, fMRI studies using the blood oxygenation level dependent (BOLD) method, perfusion PET, SPECT, and MRI using e.g. the arterial spin labeling (ASL) MRI sequence	Changes in glucose brain metabolism measured via FDG-PET is an indicator of neuronal activity [15]. SV2A-PET imaging tracers binding to the SV2A protein aims at investigating synaptic integrity [16]. Functional imaging using BOLD fMRI is an indirect measure of network dysfunction using the magnetic properties of oxygenated blood [17]. Perfusion MRI using labeling of arterial blood water as an endogenous tracer for blood flow and perfusion PET and SPECT using radiotracers [18]
Vascular disease	T1-weighted sMRI and FLAIR sequences Perfusion MRI to investigate cerebral perfusion abnormalities using dynamic contrast enhanced (DCE) MRI	WMH and enlarged perivascular spaces (PVS) are biomarkers for small vessel disease (SVD) and used to investigate the separate and joint influence of SVD and AD pathology on the disease course [19]. WMH might be of vascular or non-vascular origin [20]. Blood–brain-barrier (BBB) integrity might be reflected in cerebral perfusion abnormalities [21]
Inflammation	PET tracers 18 kDa translocator protein (TSPO) and Deuterium-L-deprenyl (DED)	TSPO-PET signal most likely reflects microglia density [22, 23], [11C]DED-PET aims to visualize activated astrocytes [24]

*Aβ* Amyloid-beta, *AD* Alzheimer’s Disease, *ASL* Arterial Spin Labeling, *BOLD* Blood Oxygenation Level Dependent, *DCE* Dynamic Contrast Enhanced, *DED* Deuterium-L-deprenyl, *DWI* Diffusion Weighted Imaging, *FBB* [18F]florbetaben, *FBP* [18F]florbetapir, *FDG* Fluorodeoxyglucose, *fMRI* functional Magnetic Resonance Imaging, *FTP* [18F]flortaucipir, *PET* Positron Emission Tomography, *PiB* Pittsburgh Compound B, *PVS* Perivascular Spaces, *sMRI* Structural Magnetic Resonance Imaging, *SPECT* Single Photon Emission Computed Tomography, *SV2A* Synaptic Vesicle Protein 2 A, *SVD* = Small Vessel Disease, *TSPO* 18 kDa Translocator Protein, *WMH* White Matter Hyperintensities

### Amyloid pathology

Neuritic Aβ plaques have long been recognized as a histopathological hallmark of AD, with early diffuse neocortical plaques depositing in the posteromedial cortex (PMC) and frontal regions. Characteristic hierarchical stages (“Thal phases”) were established by postmortem histology [6]. This staging has been largely recapitulated with Aβ-PET imaging [25], which has enabled investigating early emerging amyloidosis in cognitively normal individuals and accumulation over time. There is a spatiotemporal hierarchy of Aβ accumulation [8, 26, 27] and longitudinal Aβ progression patterns closely match cross-sectional staging [28–30]. Rates of Aβ deposition show very little variability across anatomically distant brain regions [31] and resemble sigmoid-shaped trajectories, with higher global Aβ burden at baseline predicting higher rates of neocortical Aβ accumulation in both cognitively unimpaired and impaired individuals and with accumulation slowing down at higher levels of Aβ accumulation [32, 33]. While soluble Aβ oligomers may spread across neighboring regions, plaque formation could rather depend on local factors like intense neuronal activity [34, 35].

### Tau pathology

Tau tangles are closely related to cognition [36], first deposit in the (trans)entorhinal cortex, and accumulate throughout the medial temporal lobe (MTL). In the presence of elevated Aβ, tau subsequently progresses to temporoparietal regions and finally across the neocortex. This pattern was first characterized in postmortem tissue samples [4] but has been confirmed in vivo in cross-sectional PET studies [37–39]. A priori region-based studies also suggest that tau generally accumulates in these patterns longitudinally [40, 41] but show considerable individual variability in tau deposition and spread [39, 42–45]. Tau spread along structural [46] and functional [42] connections has also been observed longitudinally. Further, higher rates of tau deposition in the MTL are predicted by locally higher baseline tau burden in cognitively unimpaired older adults and may further be driven by local activity [47]. While rates of tau accumulation were similar across brain regions in one study [48], another study reported higher rates of accumulation for temporal regions [49] in cognitively unimpaired and impaired adults. Additionally, data-driven profiling has identified fast accumulators with increased accumulation in temporal cortex and PMC [45].

### Neurodegeneration and neuronal dysfunction

Structural MRI (sMRI) has played an integral role in investigating AD progression and diagnosis. Whole-brain and hippocampal atrophy are sensitive markers of neurodegeneration and disease progression [50, 51]. At the whole-brain level, a classical "cortical signature" of AD-related atrophy is well-established and associated with symptom severity [52]. Longitudinal sMRI studies have further demonstrated that rates of gray matter loss in AD compared to elderly controls generally mirror patterns of tau accumulation [53–55] and precede symptomatic onset in both familial [56, 57] and sporadic AD [58]. However, despite these associations, atrophy is not specific for AD pathology; for example, hippocampal atrophy is associated with cognitive decline independent of Aβ and tau pathology, suggesting contributions from other pathological factors [59]. Further, studies showing increased gray matter volume or cortical thickness with early Aβ, possibly related to glia response [60], and “pseudoatrophy” in anti-amyloid trials [61, 62] call sMRI into question as being a universal marker of neurodegeneration.

FDG-PET is a widely used imaging modality for assessing region-specific aberrant brain glucose metabolism related to AD pathology. Its prognostic utility lies in its ability to detect early region-specific hypometabolism that correlates with cognitive decline before clinical symptoms become apparent [63–65]. While FDG-PET and sMRI are often used interchangeably as imaging biomarkers in AD [2], evidence from multimodal studies suggests that FDG-PET is more sensitive to early neurodegenerative processes compared to sMRI [66–68]. Moreover, the extent and pattern of hypometabolism correlate with advancing AD pathology, providing a means to track disease severity over time [69, 70] and conduct clinical classification [71, 72]. ​​Interestingly, there are differential FDG-PET patterns related to different underlying pathologies, which can provide clinically useful information for differential diagnosis [73, 74].

Modalities that target neuronal integrity, namely SV2A-PET and diffusion weighted imaging (DWI), seem to be more closely associated with tau than Aβ pathology. Longitudinal SV2A-PET studies remain scarce, but synaptic loss over time has been shown to follow tau rather than Aβ accumulation patterns [75, 76] and diffusion tensor imaging (DTI) studies have been linked to axonal integrity and show that it is particularly impacted by tau pathology, preceding both neuronal loss and clinical manifestation [77–80]. More recent advances in DWI, such as multi-shell acquisitions, allow a more detailed investigation of region-specific subtle microstructural dysfunction, providing the potential for early detection of AD [81]. Overall, however, regional onsets and spatiotemporal progression of AD-specific patterns using these modalities are still incompletely understood.

Longitudinal BOLD fMRI studies in AD typically focus on resting-state functional connectivity (FC), while longitudinal studies on task-based FC and activity are rare [17]. Using fMRI, early functional changes like “hyperactivation” and "hyperconnectivity" linked to AD pathology and cognition have been identified and are interpreted as markers of dysfunctional brain networks [82]. FMRI studies can bridge molecular and clinical research by shedding light on network mechanisms of risk and resilience to AD pathology [83–85]. However, most fMRI studies use a group approach rather than precision imaging as they were designed to contribute to cognitive neuroscience research rather than to explain between-subject variance [86, 87]. Moreover, BOLD signal changes are not specific to AD and occur in normal aging and various neurodegenerative diseases [88–90].

To summarize, MRI and FDG-PET approaches add valuable information to understand altered brain responses related to AD pathology and its progression and relationship with cognitive symptoms. However, while FDG-PET is an established marker of neurodegeneration and can be used to stage disease progression, it does not directly measure Aβ or tau pathology and therefore cannot alone determine neuropathological stage. Similarly, structural and functional MRI provide Important but indirect measures of underlying pathology. Combining these modalities with molecular imaging or other biomarkers offers a more complete and biologically specific picture of disease progression.

## Pathological interactions and potential causality

### Longitudinal characterization of the pathological cascade of Alzheimer’s disease

The classic model of AD biomarker change from normal aging along the AD continuum influenced research over the last decade greatly. It suggests that Aβ and tau accumulate up to 20 years before clinical manifestation [32, 91]. In this model, Aβ accumulation is seen as a very early, potentially initiating factor in the cascade of AD [7, 92], enabling tau spread, which in turn leads to synaptic and neuronal loss [93]. The cascading network failure model of AD [94, 95] further incorporates higher local activity of the default mode network (DMN) and higher between-network connectivity. It is debated whether these functional changes initially serve as compensatory processes for decreasing network function related to early AD pathology. However, they could also reflect oversaturation of brain networks which, in turn, leads to accelerated network failure. These complex theoretical models are largely based on cross-sectional data, and it is difficult to empirically address causality. Extensive longitudinal multimodal studies with participants from healthy adults to severe stages of AD including interventions would be critical to address the issue. Longitudinal multimodal studies have, however, contributed insights into parts of the temporal dynamics of AD.

An established finding is that Aβ drives tau accumulation and spread. Tau accumulation rates are elevated with higher Aβ burden in diverse brain areas [48]. Recent longitudinal studies showed that Aβ facilitates tau spread from medial to lateral temporal lobe and neocortical regions [47, 96, 97]. Conversely, higher baseline tau in temporal and parietal cortex was associated with faster Aβ accumulation [31].

Regarding neurodegeneration, higher superior-temporal but not global Aβ burden predicted greater cortical thinning in patients with mild cognitive impairment (MCI) but not in cognitively unimpaired adults [98]. In another study of cognitively unimpaired adults, however, higher Aβ burden at baseline predicted a steeper decline in hippocampal volume [99] and in white matter integrity of the parahippocampal cingulum, while there was no association between baseline measures [100]. Critically, longitudinal studies suggest that tau drives neurodegeneration more strongly than Aβ. Baseline global tau- but not Aβ-PET signal predicted the rate and topography of prospective atrophy in dementia patients [55]. In cognitively unimpaired older adults, the steepest rate of tau accumulation and atrophy has been reported in temporal and retrosplenial cortex, in dementia patients, however, regions differed, with the steepest rate of tau accumulation in frontal cortex and atrophy in PMC [101]. Frontotemporal cortical thinning has been found to be predicted by higher baseline tau burden, but not by change in tau-PET signal, in cognitively unimpaired and impaired individuals [102].

**Fig. 1:**
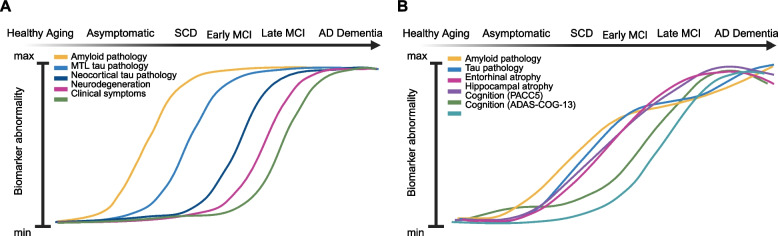
Conceptual illustration of longitudinal biomarker dynamics in Alzheimer’s disease. **A** The influential model of Jack and colleagues [2] depicts archetypical sigmoidal curves representing isolated changes in Alzheimer’s disease biomarkers over time, based on the revised AT(N) framework. Adapted from [2]. **B** We propose that moving from isolated biomarker studies to longitudinal multimodal investigations can uncover more complex interactions and causal relationships between biomarkers. The curves shown in B are adapted from a longitudinal modeling study by Lattmann-Greve and colleagues [103], illustrating how multimodal longitudinal data can reveal intricate and interacting dynamics over time. In their study, the authors utilized longitudinal CSF, MRI, and cognitive scores in a multivariate probabilistic disease progression model to generate empirical biomarker disease progression curves. The resulting curves uncovered differential hypothetically implicated biomarker trajectories with cognition being preceded by morphometry and CSF-based Alzheimer’s disease biomarkers, respectively, and different timepoints of fastest change. The authors further assessed the relationship to change in fMRI encoding task activation. These changes in activation were nonlinear and independently associated with tau positivity and neurodegeneration. Adapted from [103]

Baseline tau pathology also predicts faster synaptic loss as measured by SV2A-PET [75], and synaptic loss regionally follows tau-accumulation patterns over time [76], indicating that tau is implicated in synaptic loss. Tau pathology may also drive unfavorable functional changes. A recent study using longitudinal fMRI during encoding and cerebrospinal fluid (CSF)-markers of AD pathology proposed that MTL atrophy and tau accumulation are independently linked to reduced deactivations in the DMN, which includes the PMC [103]. Further, tau might mediate the association of Aβ and neurodegeneration [104], and conversely, Aβ might mediate the association of tau and neurodegeneration. Studies report that abnormal hippocampal cingulum bundle diffusivity at baseline predicts tau accumulation in the PMC only in Aβ-positive individuals [99]. In Aβ-positive individuals, increase in cortical tau has been further found to be related to a diffuse increase in atrophy in frontotemporoparietal areas, while increase in Aβ itself is not [49]. While the complex causal relationships along the AD cascade are still not fully understood, multimodal studies combining longitudinal biomarkers can advance our understanding of temporal dynamics beyond the current simplified models (see Fig. 1B).

### Role of network dysfunction

**Fig. 2:**
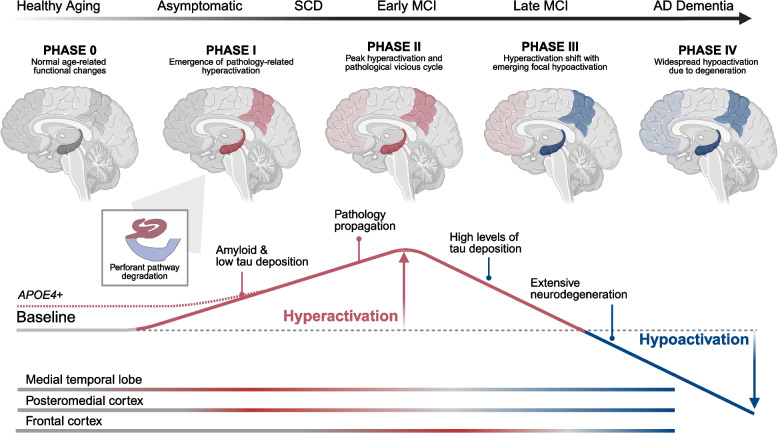
Proposed model of hyper- and hypoactivation in the Alzheimer’s disease pathological cascade. In Phase 0, non-pathological aging is characterized by functional changes (baseline, grey) in comparison with younger adults. Genetic predisposition to Alzheimer’s disease (AD) (i.e. *APOE4* genotype) may cause a prolonged state of increased activation across mid- to late life (red dotted line). In Phase I, age- and/or genetic-related functional changes predispose certain regions to pathology accumulation (i.e. hyperphosphorylated tau in medial temporal lobe (MTL) and Aβ in posteromedial cortex (PMC)). This pathology accumulation coincides with the emergence of task-based hyperactivation (red), defined as increased activation contrasted against healthy older adults, which is evident when probed with episodic memory tasks. Hyperactivation first occurs in the hippocampus, particularly within dentate gyrus/CA3, due to tau-related perforant path degeneration (see inset box) and in PMC regions due to Aβ-related effects. Overt memory impairment is not yet evident at this stage. In Phase II, disconnection between the MTL and PMC results in exaggerated hyperactivation, as well as accelerated expansion of pathology in a vicious cycle. This peak of hyperactivation is associated with SCD and early MCI. In Phase III, a tipping point of high levels of tau pathology ultimately leads to neuronal silencing and neurodegeneration, resulting in hypoactivation (blue) which first emerges in the hippocampus and PMC. Simultaneously, a shift in hyperactivation to other regions (e.g. frontal cortex) occurs. Finally, in Phase IV, widespread pathology and neurodegeneration leads to further hypoactivation that encompasses large-scale cortical regions and networks, resulting in overt cognitive impairment characteristic of AD dementia. Adapted from [17]

Network dysfunction may play a central role regarding the spatiotemporal dynamics of AD pathology. MTL and PMC hyperactivation could predispose those brain regions to pathology accumulation (i.e. tau in MTL and Aβ in PMC) and contribute to accelerated spread of pathology [17] (see Fig. 2). Further, transneuronal tau spread from the MTL to neocortical regions might be accelerated via aberrant functional connectivity [105, 106]. However, these models are largely based on animal or human cross-sectional studies, and the interplay with microstructural changes is unclear [107]. Recent longitudinal multimodal studies have begun to reveal how network changes in AD relate to pathology accumulation and spread.

Longitudinal studies in cognitively unimpaired older adults using memory task-fMRI suggest that higher and increasing BOLD signal, especially of the hippocampus, predicts the accumulation of Aβ and tau. More specifically, higher hippocampal but not frontal or occipital fMRI activation during successful encoding predicts increased accumulation of global Aβ [108] and local fMRI activity predicts increased accumulation of MTL tau [47]. Regarding the PMC, increasing precuneus activation over time during episodic retrieval relates to higher subsequent global Aβ-PET burden in *APOE4* carriers [109]. Further, increase [110] as well as decrease [111] in DMN resting-state FC (rsFC) has been related to faster Aβ accumulation, indicating failure of the DMN system as a critical precursor of spatiotemporal Aβ progression. Moreover, aberrant FC could drive tau spread. Baseline hippocampal tau predicts precuneus tau accumulation, particularly when higher rsFC between those regions and higher baseline Aβ burden are present [112]. Pathology-related higher bidirectional effective connectivity of the DMN and MTL during repetition of stimuli predicts entorhinal tau accumulation [113] and increasing within-hippocampus rsFC has been associated with plasma p-tau increase in *APOE4* carriers [114]. More specifically, tau seems to spread along functional connections. Findings from animal models show that tau spreads transneuronally from the MTL to neocortical regions [115]. Longitudinal human fMRI studies suggest the same process in humans, with aberrantly higher FC patterns accelerating tau spread [40, 105, 106, 116].

### Role of vascular disease

Although vascular dysregulation has long been acknowledged as an important contributor to AD pathology [117, 118], it is often overlooked in prevailing AD models [32]. However, longitudinal studies suggest that vascular dysregulation may be among the earliest pathological events in AD, highlighting its importance for early intervention and therapeutic development [119, 120].

Often considered a surrogate marker of small vessel disease (SVD), white matter hyperintensity (WMH) volumes have been linked to vascular dysfunction and dysregulation early in the process of AD [121]. However, emerging evidence highlights the heterogeneity of WMH pathophysiology, suggesting that WMH might also be caused by AD-related neurodegeneration and inflammation [20]*.* Longitudinal studies showed that WMH volume increase is associated with increase in Aβ-PET signal, hippocampal atrophy, and cortical thinning in elderly controls [122] and that WMH burden predicts increased hippocampal atrophy in elderly controls and MCI patients [123]. WMH progression and cortical atrophy may be mutually reinforcing processes, as individuals with higher baseline WMH volumes experience faster cortical thinning in temporal, cingulate and insular regions, and individuals with lower initial cortical thickness experience more rapid WMH progression in these regions [124]. The interplay between Aβ and WMH is complex. While Aβ deposition can exacerbate WMH burden through mechanisms like neuroinflammation and oxidative stress [125], WMH themselves may accelerate Aβ pathology by impairing clearance mechanisms [126], creating a vicious cycle amplifying pathology. A longitudinal study over eight years showed that higher WMH burden is associated with an increase in Aβ accumulation in cognitively unimpaired individuals [127]. This bidirectional relationship underscores the potential for WMH to mediate the impact of Aβ on clinical outcome, independent of traditional vascular risk factors such as hypertension.

A further biomarker for vascular contributions to AD is perivascular space (PVS) enlargement. Longitudinal studies are still rare, but recently, higher burden of cerebral microvascular lesions predicted faster progression of PVS enlargement [128]. While CSF Aβ-positivity is linked to PVS volume increase in the centrum semiovale, combined Aβ- and tau-positivity is associated with basal ganglia PVS volume increase [129].

### Role of inflammation

Aβ plaques are surrounded by activated microglia, indicating a strong relationship between the pathological progression of AD and inflammation [130–133]. Microglia migrate to Aβ lesions and are related to the degradation of Aβ peptides and the clearance of Aβ [133]. The role of microglia in causing or responding to AD pathology is still being debated [134] due to microglial cells having both protective as well as neurotoxic phenotypes [135].

To date the only confirmed visualization method of activated microglia and inflammation is PET, with cross-sectional studies using the 18kD translocator protein (TSPO) tracer [23] dominating the field. Alternative tracers are under development, such as [11C]DED-PET to assess reactive astrogliosis, which demonstrates higher binding at early stages of AD [24]. Neuroinflammation increases in AD, demonstrated by higher TSPO levels throughout the cortex, particularly in fronto-temporal regions [136]. Microglial activation is related to tau pathology and cognitive decline in symptomatic patients [137–139] but might be more closely related to Aβ burden in the absence of cognitive symptoms [140], an effect that could be modulated by the *APOE4* genotype [141]. Thus, an early peak in cortical TSPO binding might be a response to Aβ deposition, whereas a second peak in temporal regions could reflect tau propagation.

Longitudinal TSPO-PET studies have shown that neuroinflammation increases over time in AD [130, 142], correlating with cognitive impairment [142]. Increasing microglial activation over time appears to be directly related to Aβ and inversely related to glucose metabolism in AD [130]. However, neuroinflammation is a dynamic process and there might be different profiles of microglial activation that cannot be differentiated with TSPO-PET and may have a distinct impact on disease progression.

## Relationships between biomarker changes and cognition

Longitudinal Aβ-PET imaging studies have demonstrated that faster Aβ accumulation is modestly correlated with global cognitive decline over short follow-up times [143] and is linked to progression from being cognitively unimpaired to MCI over eight to ten years [110]. Recent studies also suggest that longitudinal Aβ accumulation is more closely related to changes in non-memory domains rather than episodic memory, particularly in Aβ-positive cognitively unimpaired individuals and MCI patients [143–146]. This association could be related to the tendency of Aβ to accumulate multifocally across the cortex and affect functional circuits responsible for coordinating multiple cognitive functions. Furthermore, these studies suggest that the rate of Aβ accumulation is more influential on cognitive changes at earlier clinical stages along the AD continuum. Additionally, the spatial extent of Aβ could be a more sensitive measure for cognition than Aβ levels [147]. In contrast, longitudinal tau-PET studies show that MTL and early neocortical tau accumulation are more strongly associated with episodic memory change [144] and clinical outcomes [36] than Aβ. Though this relationship is significant in adults with low Aβ burden, the association is enhanced in Aβ-positive individuals and significant regardless of concurrent atrophy. This suggests that early tau accumulation, especially when influenced by elevated Aβ, may affect cognition through mechanisms other than atrophy, such as inflammation, microstructural or metabolic changes [148–150]. Longitudinal sMRI and DWI studies have, however, shown that atrophy and microstructural changes are linked to cognition and clinical outcomes in AD [151–153]. Increases particularly in hippocampal atrophy are associated with faster decline in episodic memory in cognitively unimpaired individuals [154] and in symptomatic AD [56]. Clinical impairment is related to widespread decreases in fractional anisotropy and increases in mean diffusivity, reflecting microstructural white matter degeneration [14].

Further, metabolic and functional changes are related to cognitive decline. Longitudinal decreases in metabolism measured using FDG-PET are linked to global cognitive decline and predict cognitive instability [69, 155, 156] and decreases in ASL-measured whole-brain perfusion are related to decline in processing speed in cognitively unimpaired individuals [157]. Longitudinal fMRI studies have played a crucial role in identifying functional changes, such as specific regional activation and network connectivity patterns, that are related to early cognitive changes in AD. For example, in cognitively unimpaired individuals and Aβ-positive MCI patients, higher hippocampal activity during encoding predicts decline in global cognition [158, 159]. Similarly, the absence of hyperactivation in the precuneus during a recognition task is associated with better episodic memory performance in *APOE4* non-carriers [109]. Resting-state studies suggest early increases in connectivity between the MTL and cortical regions and the default mode network with AD pathology, which is also associated with decline in global cognition and episodic memory [88, 160, 161].

Notably, there is a more pronounced cognitive decline with vascular co-pathology. Longitudinal increase in WMH volume is steeper over the age of 60 and associated with a more rapid cognitive decline [121, 122]. Highlighting the dynamic nature of WMH, progression of WMH is related to decline, while regression and stability of WMH is related to improvement in cognition [162].

Taken together, tau accumulation is closely related to domain-specific memory decline, as well as functional changes involving the MTL-PMC episodic memory network measured with fMRI. Aβ-PET, FDG-PET, sMRI, and DWI provide valuable biomarkers to predict global cognition and clinical outcomes. However, many longitudinal cohort studies that focus on biomarkers only have a limited range of cognitive tests in their assessment, often only a coarse measure of global cognition (e.g. MMSE, MoCA) and it remains open which biomarkers can capture (future) change in more fine-grained cognitive functions.

## Biomarkers lacking current longitudinal investigation: challenges and potential future insights

**Fig. 3:**
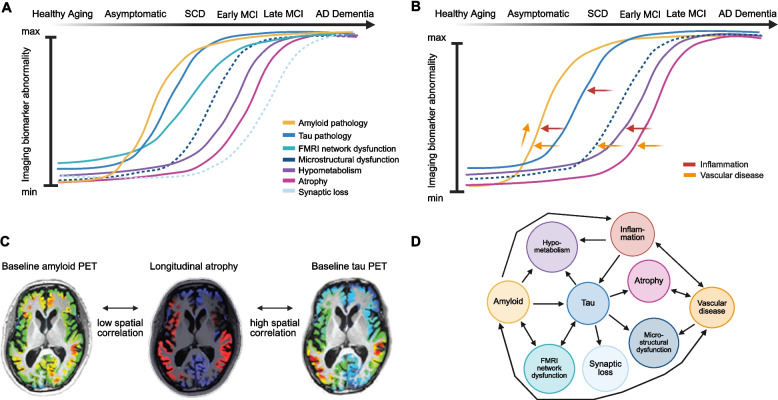
Conceptual illustration of the insights gained from longitudinal multimodal imaging biomarker studies on temporal, spatial, and causal aspects of Alzheimer’s disease pathology. This figure is not meant to be exhaustive but serves to illustrate the complex interplay of Alzheimer’s disease imaging biomarkers over time. **A** Temporal trajectories and relationships of imaging biomarkers across the disease continuum, derived from longitudinal studies discussed in this review paper. Curves depict the estimated onset, rate of change, and plateau phases for biomarkers. Dotted lines indicate biomarkers where limited longitudinal data is available. **B** Arrows depict shifts in biomarker trajectories influenced by inflammation and vascular disease, emphasizing how these additional factors alter disease trajectory. **C** Spatial correspondence of pathological processes across brain regions, illustrating patterns of co-localization and divergence as assessed by La Joie and colleagues [55] using multimodal longitudinal imaging biomarkers, can provide valuable insight into disease dynamics. Adapted from [55]. Brain plots from [55]. Reprinted with permission from AAAS. **D** Graph of causal relationships between imaging biomarkers based on studies reviewed above. Nodes represent distinct pathological processes implicated in Alzheimer’s disease. Directed edges indicate putative causal influences between processes, as estimated from longitudinal observational and experimental data to date as discussed in this review

While there is robust longitudinal data for Aβ and tau pathology that has been contributing to a better understanding of the mechanisms behind AD, other biological features, such as neuroinflammation, vascular changes, and synaptic integrity, remain underexplored, despite recent efforts and advances (see Fig. 3 for a schematic overview). These processes may present significant factors in disease progression, but it is not yet fully understood how they evolve over time. Although longitudinal studies remain the gold standard for establishing the temporal sequence of disease-related changes, emerging data-driven approaches such as SuStaIn (Subtype and Stage Inference)[163] can help infer likely progression patterns from cross-sectional datasets, providing valuable insights when longitudinal data are lacking (see [164] for a review).

A major challenge is the lack of suitable PET tracers. Sufficiently specific PET tracers for alpha-synuclein co-pathology [165, 166] are lacking, tracers such as [18F]flortaucipir bind well to 3R/4R but do not bind equally well to other tauopathies [167], and SV2A-PET assessment needs to be further validated. 11C-UCB-J is an effective PET tracer for SV2A and provides insights into synaptic density, however, it is important to recognize that it is an indirect measure of synaptic density [168, 169]. The tracer binds specifically to SV2A, a protein found in pre-synaptic vesicles, but this binding reflects the presence of synaptic vesicles rather than a direct count of synapses themselves. Longitudinal studies combining SV2A-PET with FDG-PET, fMRI and sMRI measures could generate joint topographical maps of change, contributing to a better understanding of the underlying biological processes. Advancements in tracer development can thus open up exciting new avenues for multimodal imaging research.

A second major challenge is the limited understanding of factors that accelerate AD progression and mechanisms underlying resilience and resistance. A key question regarding disease acceleration is whether vascular pathology represents an independent process or whether it is pathophysiologically connected to Aβ and tau [129, 170, 171]. Longitudinal alterations in WMH and PVS need further exploration to understand how their rate of change relates to core AD markers and cognition. Future longitudinal studies should therefore investigate the regional relationship between rate of change in WMH, Aβ and tau deposition to elucidate interactions. Further, some older adults harboring AD-pathology can stay cognitively unimpaired for longer than expected given the severity of pathology [172, 173]. Longitudinal imaging and cognitive assessment combined with post-mortem histology can shed light on mechanisms of resilience and resistance across scales [174].

A third major challenge is the lack of longitudinal data from diverse cohorts [175]. Cohorts that better reflect societal heterogeneity are crucial to better understand the complex role of socio-economic, ethno-racial and demographic factors that influence the trajectory of AD [176–178]. Further, they can pave the way to better address interindividual differences in modifiable risk factors for AD [179]. Collecting longitudinal data of diverse cohorts could therefore be a valuable aim in clinical trials for novel treatments [180]. Vice-versa, investigating these rich longitudinal datasets from clinical intervention studies can offer opportunities to infer causal relationships of disease mechanisms.

Thus, developing and validating imaging biomarkers, disentangling the contribution of co-pathologies to the trajectory of AD, and using rich datasets are central goals for future studies to better understand disease mechanisms and foster clinical advancement [181, 182].

## Conclusion

To conclude, the unique insights into AD gained from longitudinal imaging studies highlight their importance as a key direction for future research. Longitudinal human neuroimaging biomarker studies are suited to capture the temporospatial dynamics of biological changes along the Alzheimer’s continuum. By tracking changes over time, they can offer a deeper understanding of complex interacting processes like disease acceleration by co-pathology. Particularly longitudinal multimodal imaging can reveal joint evolving patterns of e.g. tau accumulation, synaptic loss and metabolic changes that cross-sectional studies cannot detect, helping to refine our understanding of disease progression and offering more accurate predictions of symptom development. Particularly when focusing on refined PET tracers and diverse cohorts, the gained insights allow for a more comprehensive perspective on the development and interplay of different pathologies, which is crucial for both early diagnosis and the evaluation of therapeutic interventions.

## Data Availability

No datasets were generated or analysed during the current study.

## Abbreviations

Aβ: Amyloid-beta
AD: Alzheimer’s Disease
ASL: Arterial Spin Labeling
BOLD: Blood Oxygenation Level Dependent
DCE: Dynamic Contrast Enhanced
DED: Deuterium-L-deprenyl
DMN: Default Mode Network
DWI: Diffusion Weighted Imaging
DTI: Diffusion Tensor Imaging
FBB: [18F]florbetaben
FBP: [18F]florbetapir
FC: Functional Connectivity
FDG: Fluorodeoxyglucose
fMRI: Functional Magnetic Resonance Imaging
FTP: [18F]flortaucipir
MTL: Medial Temporal Lobe
PET: Positron Emission Tomography
PiB: Pittsburgh Compound B
PMC: Posteromedial Cortex
PVS: Perivascular Spaces
sMRI: Structural Magnetic Resonance Imaging
SV2A: Synaptic Vesicle Protein 2A
SVD: Small Vessel Disease
TSPO: 18KDa Translocator Protein
WMH: White Matter Hyperintensities
